# A survey of UK standards of radiation protection amongst orthopaedic surgeons

**DOI:** 10.1093/bjr/tqaf162

**Published:** 2025-07-16

**Authors:** George Ninkovic-Hall, Anna Chapman, Athanasios Saratzis, Raghu Lakshminarayan, Dan Carradice, Kaji Sritharan

**Affiliations:** Department of Vascular Surgery, Lancashire Teaching Hospital NHS Foundation Trust, Preston, Lancashire, PR2 9HT, United Kingdom; Department of Trauma & Orthopaedic Surgery, University Hospitals Coventry & Warwickshire NHS Trust, Coventry, CV2 2DX, United Kingdom; Department of Cardiovascular Sciences, NIHR Leicester Cardiovascular Biomedical Research Centre, Leicester, LE3 9QP, United Kingdom; Hull University Teaching Hospitals NHS Trust, Hull, HU3 2JZ, United Kingdom; Hull University Teaching Hospitals NHS Trust, Hull, HU3 2JZ, United Kingdom; Hull York Medical School, Hull, HU6 7RU, United Kingdom; Department of Surgery & Cancer, Faculty of Medicine, Imperial College London, W12 0NN, United Kingdom

**Keywords:** radiation protection, orthopaedic surgery, personal protective equipment, dosimetry, ALARA

## Abstract

**Objective:**

To evaluate training in radiation protection, awareness of local policies, and current practices regarding safe working with ionizing radiation among UK orthopaedic surgeons.

**Methods:**

A 37-question online survey was distributed to UK orthopaedic resident doctors and consultants through social media platforms. The survey assessed demographics, use of radiation-guided procedures, training, knowledge of safety policies, access to and use of personal protective equipment (PPE), and monitoring of radiation exposure. Data were analysed using Stata software and Pearson’s Chi-squared test.

**Results:**

Twenty-eight consultants and 79 resident doctors responded, comprising 0.4% and 5.3% of the orthopaedic workforce, respectively. Consultants were more likely to have completed formal radiation safety training (93% vs 38%; *P* < .001) and were more aware of local safety policies (56% vs 9%; *P* < .001). Access to dosimeters was limited (32% of consultants vs 6% of resident doctors; *P* < .005), with few receiving exposure feedback (20% of consultants vs 3% of resident doctors; *P* < .005). Awareness and application of the “as low as reasonably achievable” principles were poor, with 33% of resident doctors unfamiliar compared to 4% of consultants (*P* < .005). PPE use was inconsistent; 64% of consultants and 41% of resident doctors never used radiation protection glasses, and only 12% of consultants and 1.4% of resident doctors had custom-fitted lead aprons (*P* < .05).

**Conclusion:**

This study underscores deficiencies in radiation protection for UK orthopaedic surgeons, particularly resident doctors, highlighting the urgent need for mandatory radiation safety training, improved PPE provision, and monitoring of radiation exposure with regular exposure feedback.

**Advances in knowledge:**

This survey identifies deficiencies in radiation safety training and PPE access among UK orthopaedic surgeons, particularly resident doctors. It highlights the lack of substantial improvements since previous studies, underlining the need for high level systemic changes. The survey advocates for mandatory radiation safety training, consistent monitoring of radiation exposure, and the desire for the establishment of a national registry to record an individual’s annual exposure to radiation.

## Introduction

The significant and increasing need for intraoperative fluoroscopy in orthopaedic surgery, particularly trauma procedures such as open reduction internal fixation (ORIF), spinal surgery, and joint injection, has led to heightened occupational radiation exposure, posing potential long-term health risks for orthopaedic surgeons.^1^^,^^2^ In the United Kingdom, 92% of resident doctors reported using intraoperative ionizing radiation at least once per week.^3^

Despite available protective measures, such as, lead aprons, lead glasses and thyroid shields, which when used correctly, have been proven to provide sufficient protection against radiation exposure during procedures using fluoroscopy in orthopaedic theatres,^1^ studies have revealed their inconsistent use and variable awareness of radiation safety practices amongst clinicians.^2^^,^^3^ Longstanding research, emphasizes the critical need for enhanced radiation safety, especially considering the risks of cancers and non-cancerous conditions, such as, cataracts associated with cumulative radiation exposure.^4^^,^^5^ Notably, the risk of breast cancer is heightened for female orthopaedic surgeons due to inadequate protective coverage for sensitive tissues.^1^^,^^6^ The need to improve breast tissue protection has been the focus of a recent campaign by the British Orthopaedic Association (BOA).^7^

The aim of this survey was to evaluate whether improvements have been made from previous studies with respect to knowledge of radiation protection, access to personal protection equipment (PPE) and monitoring of radiation exposure amongst UK orthopaedic surgeons.

## Methods

A validated 37-question online survey was designed by a group of consultant vascular surgeons, interventional radiologists, and orthopaedic surgeons. The survey was divided into eight sections: specialty, ionizing procedures performed, demographic data, beliefs and values, training in radiation protection, strategies employed to reduce ionizing radiation exposure, access to PPE, and injuries potentially associated with working with ionizing radiation. A mixture of open and closed questioning was used, with all closed questions mandatory. All responses were self-reported and fully anonymized, with no identifiable data such as institution or geographical region collected. Pre-testing and review for content and construct validity was performed by three vascular surgeons, one interventional radiologist, and three orthopaedic surgeons.

The survey was distributed to orthopaedic resident doctors and consultants across the United Kingdom via social media and regional orthopaedic training groups. Responses were collected over a six-week period using SurveyMonkey (Momentive Inc., San Mateo, California, USA). Participation was voluntary, and respondents provided informed consent prior to starting the survey. The study was conducted as a service evaluation involving healthcare professionals, and therefore, formal ethical approval was not required. Data was analysed using Stata 18.0 (StataCorp LLC, College Station, Texas, USA), and the results were presented as descriptive statistics with Pearson’s Chi-squared tests used to assess associations between groups. As this was a descriptive cross-sectional survey intended to explore practice variation and awareness, no formal sample size calculation was performed.

## Results

### Demographics

The survey was completed by 28 orthopaedic consultants and 79 resident doctors, representing approximately 0.4% of the consultant workforce and 5.3% of the resident doctor workforce in the United Kingdom. Sixty-eight percent (19/28) of orthopaedic consultants reported biological sex as male, and 18% (5/28) identified as female. Among the orthopaedic resident doctors, 73 were registrars (92%), 3 SAS doctors (3.8%), and 3 fellows (3.8%). Of the 84% (66/79) that reported their biological sex, 46% (36/79) were male and 37% (29/79) were female.

### Procedures performed and setup

C-arm-guided trauma procedures, including intramedullary nailing and ORIF, were the most undertaken procedures, performed by 75% of consultants and 94% of resident doctors (*P* < .05). A greater proportion of resident doctors also performed elective osteotomies and joint fusions (89% of resident doctors vs 68% of consultants; *P* < .05), and joint injections in theatre (80% of resident doctors vs 64% of consultants; *P* = .1). Consultants reported higher involvement in spinal procedures, including trauma fixation (56% of consultants vs 14% of resident doctors; *P* < .0005) and elective spinal fusion (54% of consultants vs 18% of resident doctors; *P* < .005).

Resident doctors reported more frequent use of mini C-arm fluoroscopy across several domains compared to consultants, including extremity trauma (77% vs 50%; *P* < .05), elective procedures (63% vs 36%; *P* < .05), and extremity injections (58% vs 32%; *P* < .05).

### Radiation risk awareness

#### Concerns

**Figure 1.**
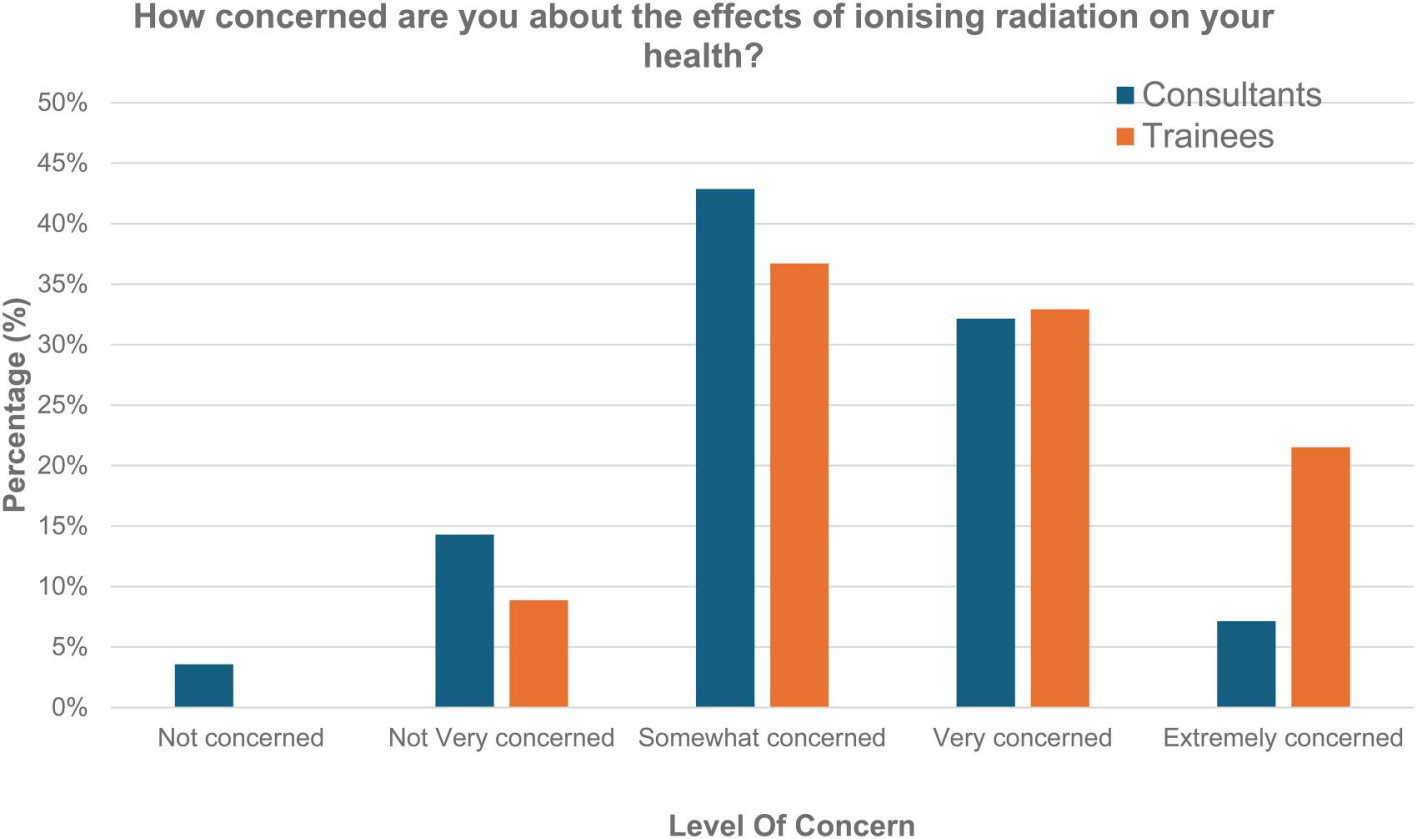
Self-reported levels of concern regarding ionizing radiation.

Figure 1 illustrates the self-reported levels of concern regarding ionizing radiation. A higher proportion of resident doctors than consultants reported being either “very concerned” or “extremely concerned” about the effects of ionizing radiation on their health (54% vs 39%, respectively; ns). The most common response in both groups was “somewhat concerned” (43% of consultants’ vs 37% of resident doctors; ns). Only a small minority reported being “not at all” or “minimally concerned” (3.6% of consultants’ vs 9% of resident doctors; ns).

#### Radiation safety training

The majority of consultants (93%) had undergone formal radiation safety training, 4% had not and 4% were unable to recall if they had. This was compared to only 38% of resident doctors (*P* < .001) who had undergone training, with 44% not trained, and 18% who could not remember.

Resident doctors were more likely to complete e-learning courses (67% vs 72% of consultants; ns) and less likely to participate in face-to-face courses (30% vs 56% of consultants) or hands-on training (7% vs 16% of consultants). Twenty-three percent of resident doctors and 16% of consultants had completed their training within the last year. Thirty-two percent of consultants compared to 3% of resident doctors had trained over 10 years ago (*P* < .005).

### Awareness of local policy

Consultant awareness of local radiation policy and contacts exceeded that of resident doctors. Sixty-three precent knew their Trust’s radiation protection officer (RPO) contact details, compared to just 10% of resident doctors (*P* < .001), and 44% of consultants had met their RPO versus only 6% of resident doctors (*P* < .001).

Consultants were also more aware of pregnancy-related radiation policy, with 33% of consultants fully aware compared to only 14% of resident doctors (*P* < .05). Similarly, 56% of consultants were fully aware of local radiation safety policy compared to only 9% of resident doctors (*P* < .00001). Overall, 11% of consultants were completely unaware of local radiation safety policy, compared to 43% of resident doctors (*P* < .05).

### Methods to reduce radiation exposure

**Figure 2.**
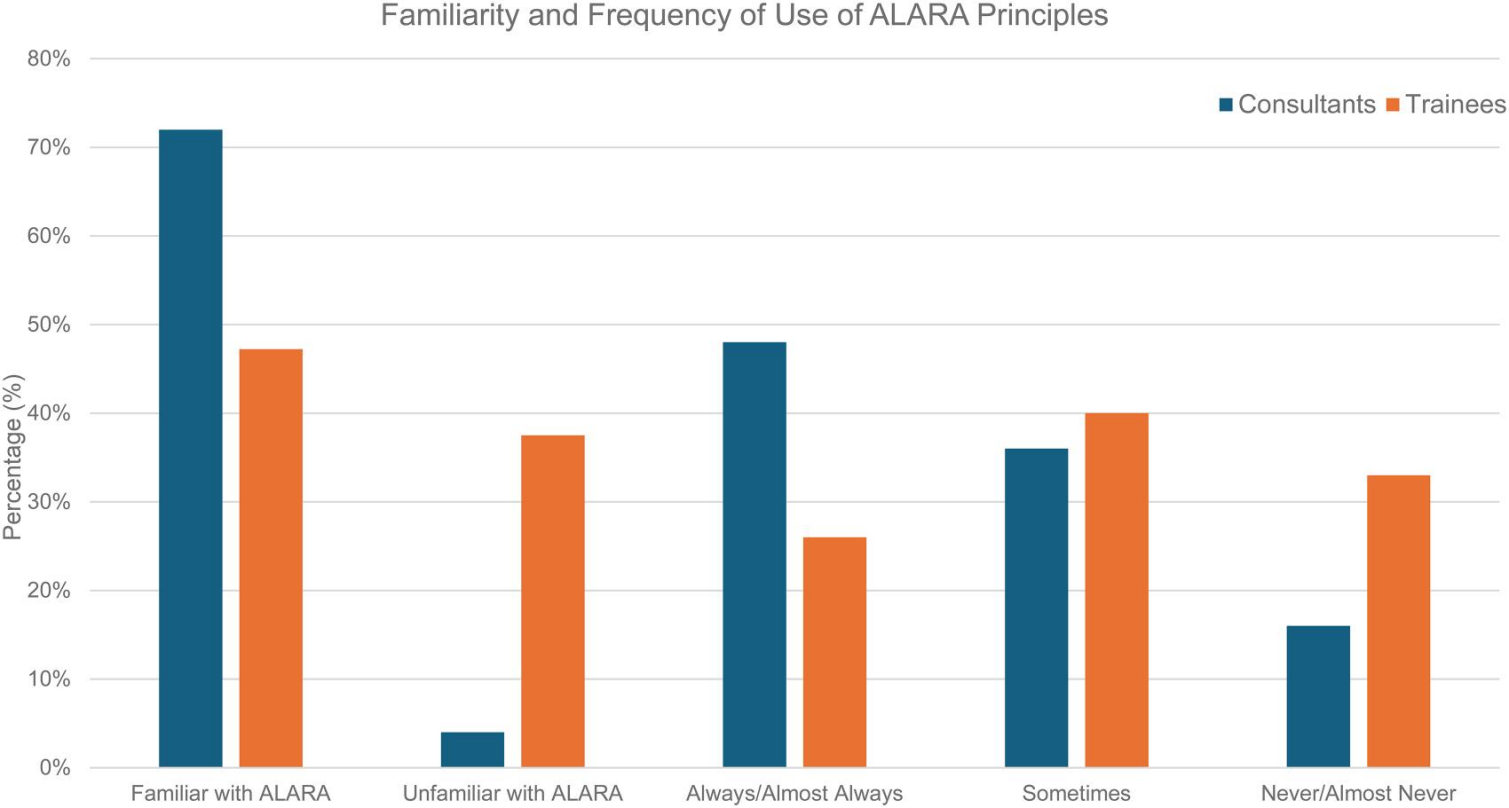
Familiarity and frequency of use of ALARA principles.

Familiarity with ALARA (As Low As Reasonably Achievable) principles was significantly higher among consultants (72%) than resident doctors (47%), with 38% of resident doctors unfamiliar with ALARA entirely, compared to 4% of consultants (*P* < .005) (Figure 2).

Consistent application of ALARA strategies (“always” or “almost always”) was more frequently reported by consultants than resident doctors (48% vs 26%, respectively; *P* < .05). Inconsistent or no use (“never” or “almost never”) was higher among resident doctors (33% vs 16% of consultants; *P* < .05). Both groups reported similar rates of occasional use of ALARA principles (36% of consultants and 40% of resident doctors; ns) (Figure 2).

**Figure 3.**
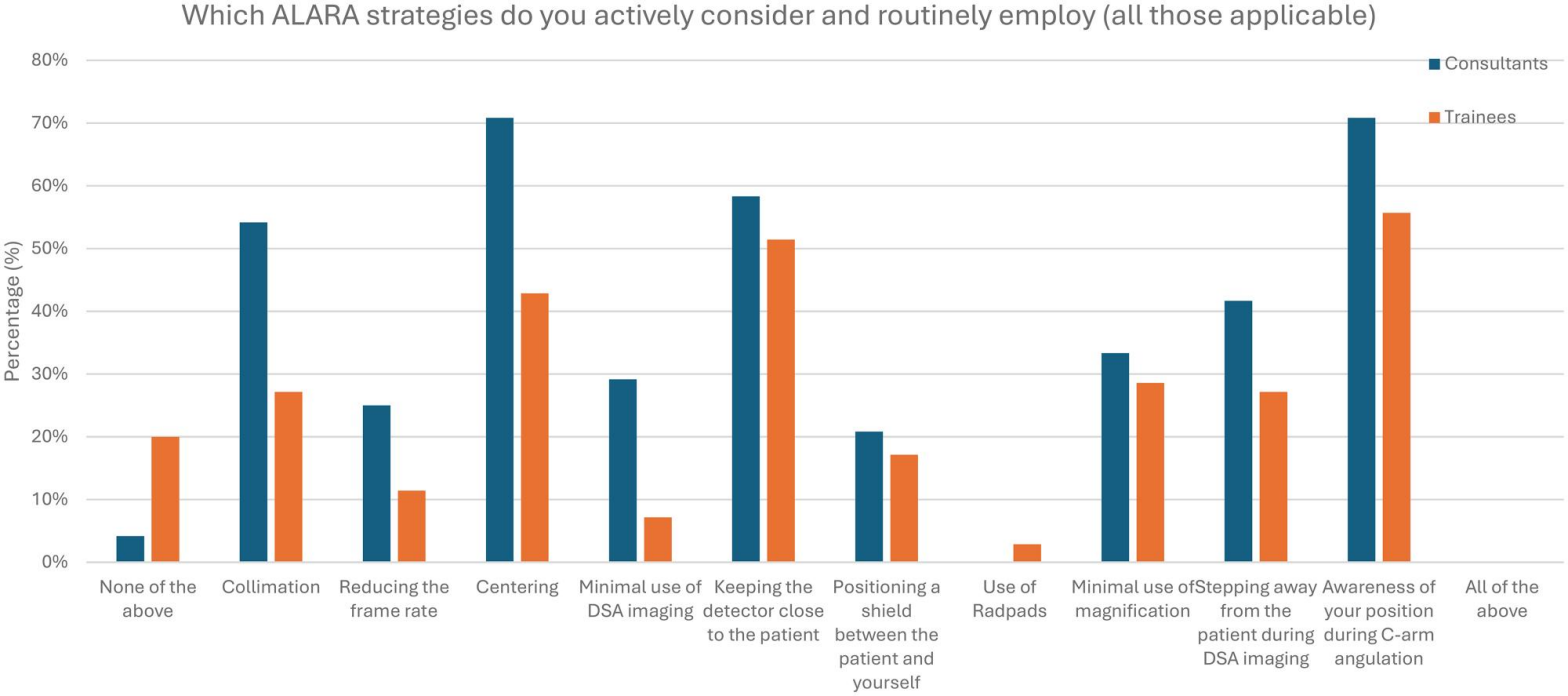
ALARA strategies actively considered and routinely employed.

Consultants compared to resident doctors more frequently employed specific ALARA techniques such as collimation (54% vs 27%, respectively; *P* < .05), centering (71% vs 43%, respectively; *P* < .05), and limiting the use of DSA imaging (29% vs 7%, respectively; *P* < .05). Other techniques, such as minimizing magnification, maintaining detector proximity, or stepping away during DSA, showed smaller differences between groups. Importantly, 20% of resident doctors did not actively use any of the above ALARA strategies, compared to just 4% of consultants (*P* < .05; Figure 3).

### Personal protective equipment

#### Lead gowns and thyroid collars

Only 12% of consultants and 1.4% of resident doctors had custom-fitted lead aprons (*P* < .05), and few had been measured for an appropriately fitting standard apron (9.1% vs 1.4%; *P* < .05). Annual apron inspections were more frequently reported by consultants than resident doctors (36% vs 13%, respectively; *P* < .05), though most respondents were unsure if checks occurred. Thyroid collar usage differed markedly between groups with 36% of consultants reporting never using them, compared to just 3% of resident doctors (*P* < .00005). Consistent use (100% of the time) was reported by 32% of consultants and 36% of resident doctors. Additionally, 4% of resident doctors indicated that thyroid collars were not provided.

#### Eye protection and PPE access

Eye protection usage was poor across both groups. 64% of consultants and 41% of resident doctors reported never using radiation protection glasses, with only 4% and 3%, respectively, consistently using them (100% of the time). Intermediate use (25%-75% of the time) was also low at just 4% in both groups. Provision was a key barrier to their use with 20% of consultants and 52% of resident doctors stating that glasses were not available (*P* < .05). Access to other protective equipment was similarly limited. Mobile or fixed lead shielding was unavailable to 16% of consultants and 36% of resident doctors (*P* < .005).

Resident doctors were more likely to request customized lead aprons than consultants (38% vs 21%) and eye protection (14% vs 8%). Management would deny resident doctors their request for PPE most often due to their resident doctor status (55%) or temporary roles (31%); while consultants most often received no explanation for refusal (73%).

### Monitoring dose exposure

#### Dosimeter availability and feedback

Only 32% of consultants and 6% of resident doctors reported having a dosimeter (*P* < .005). Among those traveling to remote sites, 40% of consultants and 57% of resident doctors lacked a dedicated dosimeter. Of those that had dosimeters, only 20% of consultants and 3% of resident doctors had been given feedback on their radiation exposure.

### Incidence and awareness of radiation-associated health conditions

Potential radiation-associated conditions were reported by 29% of consultants and 53% of resident doctors, with the latter more frequently experiencing orthopaedic complaints (41% of resident doctors vs 17% of consultants; *P* < .05); erythema/dermatitis (18% of resident doctors vs 4% of consultants), and hair loss (9% of resident doctors vs 4% of consultants). Resident doctors were also more likely to know someone who had been affected by potentially radiation-associated cancers, including breast cancer (33% of resident doctors’ vs 17% of consultants) and thyroid cancer (21% of resident doctors vs 8% of consultants).

### Perceptions of radiation protection and monitoring

Most consultants (67%) and resident doctors (86%) felt radiation protection for healthcare staff was inadequate. Confidence in employers’ monitoring was similarly low; 67% of consultants and 77% of resident doctors had no confidence.

Support for a national registry was high: 63% of consultants and 80% of resident doctors supported annual individual central dose recording and 83% of resident doctors and 63% of consultants backed cumulative lifetime tracking of radiation exposure. 89% of resident doctors but only 67% of consultants favoured a mechanism to centrally record health conditions which could be associated with occupational exposure to ionizing radiation (*P* < .05).

## Discussion

This national survey provides insights into current practices, awareness, and attitudes regarding radiation protection among UK orthopaedic surgeons. In the United Kingdom the Ionizing Radiation Regulations 2017 (IRR17), enforced by the Health and Safety Executive (HSE), establishes the safety standards for workers exposed to ionizing radiation in the workplace. These regulations mandate compliance with ALARA principles through a combination of safety practices, engineering controls, and adequate PPE. Despite these regulatory frameworks and professional guidance in existence, substantial gaps persist regarding radiation protection, particularly among resident doctors in orthopaedic surgery.

### Training and ALARA strategy awareness

Resident doctors were significantly less likely than consultants to have received formal training in radiation protection (38% vs 93%; *P* < .001), and this is likely to have impacted on their lower familiarity with ALARA principles compared to consultants (4% compared to 38%, respectively; *P* < .005) and potentially explains why a smaller proportion of resident doctors (26% vs 48% of consultant) consistently applied ALARA strategies, with 33% reporting never or almost never applying them. These results echo those of the 2021 RIO study,^3^ which reported similar deficiencies in training and protective equipment uptake among orthopaedic surgeons. The study concluded that a lack of standardized training placed both patients and healthcare professionals at increased risk, recommending the implementation of a national training programme to address these gaps.

Multiple studies have also shown that less experienced orthopaedic surgeons tend to rely more heavily on fluoroscopy, resulting in higher radiation exposure compared to their senior counterparts.^8–11^ This again highlights the need for role-specific, mandatory national training courses^12^ to improve knowledge of radiation protection strategies and the use of strategies to minimize radiation exposure. The International Commission on Radiological Protection (ICRP) advocate for training to start during medical school and to be updated every 36 months.^13^ Implementing a training programme of this nature could address the initial gaps in both training and practical application, significantly enhancing radiation safety for resident doctors and consultants.

While our study did not capture the duration or specific learning objectives of radiation safety training undertaken, the limited uptake and heavy reliance on e-learning, particularly among resident doctors (68%) raises concerns regarding the adequacy of this format of training. The best method of radiation protection training delivery requires further evaluation.

### PPE use and radiation-associated health risks

Orthopaedic surgeons can face annual radiation exposure levels ranging from 0.5 to 3.5 mSv, with inadequate use of protective equipment, such as poorly fitted lead aprons, potentially increasing their exposure by up to 30%.^14^ Previous studies have demonstrated that less than 50% of orthopaedic surgeons consistently use thyroid shields.^3^^,^^15^^,^^16^ Our study again reveals inconsistent PPE use. Only 2% of resident doctors and 12% of consultants had custom-fitted lead aprons. Radiation protection glasses were never used by 64% of consultants and 41% of resident doctors. Similarly, 36% of consultants and 2.9% of resident doctors never wore thyroid collars. These findings are concerning given the well-documented risks of thyroid cancer,^16^ breast cancer,^17^^,^^18^ brain tumours,^19^ other malignancies,^20^ and cataracts^21^^,^^22^ associated with radiation exposure. Importantly, a lack of access or provision was often cited as a reason for not wearing PPE and this is simply not acceptable. Erythema, dermatitis, and hair loss among healthcare workers suggests potential under-protection during radiation-guided procedures.^23^ The European Society for Vascular Surgery (ESVS) 2023 Clinical Practice Guidelines on Radiation Safety, affirms that skin injuries are common deterministic effects of radiation exposure and markers of inadequate shielding or procedural protection.^24^ In our study, 29% of consultants and 53% of resident doctors reported experiencing conditions which could be related to their occupational exposure to radiation. However, these outcomes were self-reported, and therefore, must be interpreted cautiously as they have not been independently clinically verified.

### Dosimetry gaps, risk assessments, and monitoring practice

Understanding and monitoring radiation dose exposure is critical to ensuring occupational safety in operative environments. Current guidance from the HSE and the IRR17 sets annual dose limits at 20 mSv, with a five-year cumulative cap of 100 mSv, and no single year to exceed 50 mSv.^25^ Despite these thresholds, only 32% of consultants and 6% of resident doctors reported having access to a dosimeter, with even fewer receiving feedback on their annual radiation dose exposure (20% of consultants vs 3% of resident doctors, respectively). This lack of consistent monitoring and feedback undermines both personal awareness and institutional safety efforts.

Critically, whilst on one hand the Ionizing Radiation (Medical Exposure) Regulations (IRMER) and IRR17 mandate employers to provide radiation dose monitoring for staff classified as exposed workers, on the other hand, current IRR17 guidance also allows local risk assessments to determine whether dosimetry is necessary. If estimated exposure remains below 1 mSv/year,^26^ dosimeters may not be mandated. However, in a modern orthopaedic setting with widespread use of fluoroscopy and frequent hospital rotation among resident doctors, such discretion may contribute to under-monitoring. A disconnect between Radiation Protection Adviser (RPA) recommendations and implementation may further exacerbate the issue. Although RPAs are formally required to guide local risk assessments and PPE policy, only 10% of resident doctors and 63% of consultants knew how to contact their local RPA, with even fewer having met them.

#### Monitoring rotational doctors

There is no national standardized approach to reconcile cumulative dose recording for staff rotating between Trusts.^27^ In this context, a single, wearable personal dosimeter, especially for resident doctors and locum doctors, may offer more consistent and reliable long-term exposure tracking.^14^ For substantive consultants, institution-specific monitoring may suffice, but a hybrid model with cross-compatible systems may be most appropriate. Whichever model is adopted, regular feedback must accompany dosimeter use to reinforce good behaviours.

### Support for a national radiation dose registry

While current legislation under IRMER and IRR17 mandates that employers monitor and record radiation exposure, our data show substantial inconsistencies in practice, particularly among resident doctors. A national registry for annual and lifetime radiation dose monitoring may offer a pragmatic solution to address these gaps, particularly for clinicians who rotate across multiple institutions. Most resident doctors (80%) and consultants (63%) supported a national registry to record individual annual radiation dose exposures, with similar numbers favouring a national database to track lifetime cumulative radiation exposure (83% and 63%, respectively). A registry for radiation-related health conditions was also widely supported, particularly by resident doctors (89% vs 67% of consultants). The potential value of a national registry is increasingly recognized. Systems such as Canada’s National Dose Registry demonstrates the feasibility of secure, centralized tracking of cumulative occupational exposure.^24^ Such a registry could standardize monitoring across Trusts, provide automated alerts at threshold levels, and facilitate epidemiological research into radiation-associated health risks. Concerns regarding data protection and punitive “league tables” could be mitigated by anonymized or pseudonymized records, GDPR compliance, and robust ethical oversight. Instead of being a regulatory stick, such a system could offer early warning mechanisms and support targeted interventions.

### Limitations

This study has some limitations. The sample size is small, and therefore, this study may not necessarily be reflective of practice in this group. There is also the potential for responder bias, as participants may be those who have heightened concerns about radiation exposure. Additionally, institution or regional variation were not captured, preventing evaluation of geographic influences. Despite these limitations, our findings align with existing literature, confirming significant, unresolved concerns about radiation safety in UK orthopaedic surgery.

## Conclusion

Despite previous calls for improvement, our findings reaffirm critical gaps in radiation safety among orthopaedic surgeons in the United Kingdom, particularly resident doctors in orthopaedic surgery. Addressing these gaps will require structured, mandatory training, consistent PPE access, regular dosimeter feedback, improved compliance and engagement from the orthopaedic workforce and comprehensive monitoring of occupational radiation exposure. The widespread support for a national registry to track both annual exposure and radiation-associated health conditions further highlights the need for transparent, rigorous safety protocols to protect healthcare professionals in orthopaedic surgery.

## References

[1] Hurley RJ , McCabe FJ, Turley L, Maguire D, Lucey J, Hurson CJ. Whole-body radiation exposure in trauma and orthopaedic surgery. Bone Joint Open. 2022;3:907-912. 10.1302/2633-1462.311.Bjo-2022-0062.R1 36416077 PMC9709492

[2] Bahari S , Morris S, Taylor C, et al The hot zone—the need for collar and cuffs? PROSPECTIVE study of radiation exposure in hands and thyroid during wrist and hand procedure. Orthop Proc. 2006;88-B:286-286. 10.1302/0301-620X.88BSUPP_II.0880286d

[3] Raza M , Geleit R, Houston J, Williams R, Trompeter A. Radiation in orthopaedics (RIO) study: a national survey of UK orthopaedic surgeons. Br J Radiol. 2021;94:20210736. 10.1259/bjr.20210736 34235964 PMC9327761

[4] Stoker D. Ionising radiation and the orthopaedic surgeon. J Bone Joint Surg Br. 1992;74:934-934. 10.1302/0301-620x.74b6.1447264 1447264

[5] Blachut P. Radiation exposure in orthopaedic trauma surgery. Orthop Proc. 2008;90-B:139. 10.1302/0301-620X.90BSUPP_I.0880139b

[6] Pilkington I , Sevenoaks H, James E, Eastwood D. Protecting female healthworkers from ionising radiation at work. BMJ. 2023;381:e075406. 10.1136/bmj-2023-075406 37045449

[7] Sevenoaks H. A Tipping Point to Improve Radiation Safety in Orthopaedics? British Orthopaedic Association. Accessed April 2023. https://www.boa.ac.uk/resource/a-tipping-point-to-improve-radiation-safety-in-orthopaedics.html

[8] Malik AT , Rai HH, Lakdawala RH, Noordin S. Does surgeon experience influence the amount of radiation exposure during orthopedic procedures? A systematic review. Orthop Rev (Pavia). 2019;11:7667. 10.4081/or.2019.7667 30996838 PMC6452094

[9] Quah C , Mehta R, Shivji FS, et al The effect of surgical experience on the amount of radiation exposure from fluoroscopy during dynamic hip screw fixation. Ann R Coll Surg Engl. 2017;99:198-202. 10.1308/rcsann.2016.0282 27551896 PMC5450269

[10] Magee LC , Karkenny AJ, Nguyen JC, et al Does surgical experience decrease radiation exposure in the operating room? J Pediatr Orthop. 2021;41:389-394. 10.1097/bpo.0000000000001825 34096557

[11] Rashid MS , Aziz S, Haydar S, Fleming SS, Datta A. Intra-operative fluoroscopic radiation exposure in orthopaedic trauma theatre. Eur J Orthop Surg Traumatol. 2018;28:9-14. 10.1007/s00590-017-2020-y 28798994 PMC5754436

[12] Raza M , Houston J, Geleit R, Williams R, Trompeter A. The use of ionising radiation in orthopaedic surgery: principles, regulations and managing risk to surgeons and patients. Eur J Orthop Surg Traumatol. 2021;31:947-955. 10.1007/s00590-021-02955-9 33825954

[13] Vañó E , Rosenstein M, Liniecki J, Rehani MM, Martin CJ, Vetter RJ. ICRP publication 113. Education and training in radiological protection for diagnostic and interventional procedures. Ann ICRP. 2009;39:7-68. 10.1016/j.icrp.2011.01.002 21788173

[14] Rowantree SA , Currie C. Orthopaedic surgeons’ knowledge and practice of radiation safety when using fluoroscopy during procedures: a narrative review. Radiography. 2024;30:274-281. 10.1016/j.radi.2023.11.017 38041915

[15] Wan RCW , Chau WW, Tso CY, et al Occupational hazard of fluoroscopy: an invisible threat to orthopaedic surgeons. J Orthop Trauma Rehabil. 2021;28:22104917211035547. 10.1177/22104917211035547

[16] Iglesias ML , Schmidt A, Ghuzlan AA, et al Radiation exposure and thyroid cancer: a review. Arch Endocrinol Metab. 2017;61:180-187. 10.1590/2359-3997000000257 28225863 PMC10118869

[17] John EM , Phipps AI, Knight JA, et al Medical radiation exposure and breast cancer risk: findings from the breast cancer family registry. Int J Cancer. 2007;121:386-394. 10.1002/ijc.22668 17372900

[18] Chou LB , Johnson B, Shapiro LM, et al Increased prevalence of breast and all-cause cancer in female orthopaedic surgeons. JAAOS Glob Res Rev. 2022;6:e22.00031. 10.5435/JAAOSGlobal-D-22-00031 PMC9126513 35587823

[19] Roguin A , Goldstein J, Bar O. Brain tumours among interventional cardiologists: a cause for alarm? Report of four new cases from two cities and a review of the literature. EuroIntervention. 2012;7:1081-1086. 10.4244/eijv7i9a172 22207231

[20] Ron E. Ionizing radiation and cancer risk: evidence from epidemiology. Radiat Res. 1998;150:S30-S41. 9806607

[21] Alhasan AS , Aalam WA. Eye lens opacities and cataracts among physicians and healthcare workers occupationally exposed to radiation: a systematic review and meta-analysis. Saudi Med J. 2022;43:665-677. 10.15537/smj.2022.43.7.20220022 35830987 PMC9749701

[22] Della Vecchia E , Modenese A, Loney T, et al Risk of cataract in health care workers exposed to ionizing radiation: a systematic review. Med Lav. 2020;111:269-284. 10.23749/mdl.v111i4.9045 32869764 PMC7809955

[23] Wiper A , Katira R, Roberts DH. Images in cardiology. Interventional cardiology: it’s a hairy business. Heart. 2005;91:1432. 10.1136/hrt.2005.064899 16230441 PMC1769174

[24] Modarai B , Haulon S, Ainsbury E, et al Editor’s choice—European Society for Vascular Surgery (ESVS) 2023 clinical practice guidelines on radiation safety. Eur J Vasc Endovasc Surg. 2023;65:171-222. 10.1016/j.ejvs.2022.09.005 36130680

[25] Association BO. Occupational Radiation Exposure Risk in Orthopaedics. https://www.boa.ac.uk/standards-guidance/radiation-exposure-in-theatre/t-and-o/occupational-radiation-exposure-risk.html?_gl=1*x5ccka*_up*MQ.*_ga*MjEzMDI2NDY5NC4xNzMzNzc4ODQ1*_ga_GZM76H2EF6*MTczMzc3ODg0NC4xLjEuMTczMzc3ODg3Ni4wLjAuMA

[26] Executive HaS. *Working with Ionising Radiation. Ionising Radiations Regulations 2017. Approved Code of Practice and guidance. L121*. 2nd ed. Vol. 2. TSO (The Stationery Office); 2018.

[27] Rogers A , Chapple CL, Murray M, Platton D, Saunderson J. UK guidance on the management of personal dosimetry systems for healthcare staff working at multiple organizations. Br J Radiol. 2017;90:20170363. 10.1259/bjr.20170363 28936897 PMC5963377

